# The effect of adjuvant chemotherapy on survival in Korean patients with node negative T1c, triple negative breast cancer

**DOI:** 10.1371/journal.pone.0197523

**Published:** 2018-05-16

**Authors:** Seung Taek Lim, Chan Heun Park, Sung Yong Kim, Seok Jin Nam, Eun Young Kang, Byung-In Moon, Hyouk Jin Lee, Ye Won Jeon, Hongki Gwak, Young Jin Suh

**Affiliations:** 1 Department of Surgery, Division of Breast & Thyroid Surgical Oncology, College of Medicine, St. Vincent’s Hospital, The Catholic University of Korea, Suwon, Republic of Korea; 2 Department of Surgery, Kangbuk Samsung Hospital, Sungkyunkwan University School of Medicine, Seoul, Republic of Korea; 3 Department of Surgery, Soonchunhyang University Cheonan Hospital, Soonchunhyang University College of Medicine, Cheonan, Korea; 4 Department of Surgery, Division of Breast and Endocrine Surgery, Samsung Medical Center, Sungkyunkwan University School of Medicine, Seoul, Korea; 5 Department of Surgery, Seoul National University College of Medicine, Seoul National University Bundang Hospital, Seongnam, Korea; 6 Department of Surgery, Ewha Womans University School of Medicine, Seoul, Korea; 7 Department of Surgery, Saegyaero Hospital, Busan, Republic of Korea; University of Nebraska Medical Center, UNITED STATES

## Abstract

**Background:**

The present study investigated the prognostic role of adjuvant systemic chemotherapy in patients with node negative, T1c triple negative breast cancer (TNBC) from a nationwide cohort. In addition, the prognostic effect between 3 different chemotherapy regimens were compared in node-negative T1c TNBC patients by subgroup analysis.

**Methods:**

From the Korean breast cancer registry database, 1,151 T1c node negative TNBC patients were included in this study. Patients were categorized into four treatment groups according to chemotherapy regimen: (1) no chemotherapy, (2) adriamycin plus cyclophosphamide (AC), (3) adriamycin/epirubicin plus cyclophosphamide plus 5-FU (FAC/FEC), and (4) cyclophosphamide plus 5-FU plus methotrexate (CMF). Overall survival (OS) was evaluated between each patient group.

**Results:**

Of the 1,151 T1c node negative TNBC patients, 1,006 received adjuvant chemotherapy, while 145 received no chemotherapy. Among the patients receiving adjuvant chemotherapy the distribution of regimens was: 586 AC, 168 FAC/FEC (126 FAC, 42 FEC), and 252 CMF. The mean follow-up time of the full study cohort was 87.98 ± 33.56 months (range = 6–192 months). Patients in the no chemotherapy group showed significantly worse OS compared to each chemotherapy regimen group. However, when OS was compared between each chemotherapy regimen, no significant difference was found.

**Conclusions:**

This study showed that adjuvant systemic chemotherapy improved OS in T1c node negative TNBC patients, regardless of chemotherapy between AC, FAC/FEC, and CMF regimens.

## Introduction

Triple negative breast cancer (TNBC) is defined as hormone receptor negative and HER2 receptor negative breast cancer, which comprises approximately 10% to 25% of breast cancer molecular subtypes [1–3]. Due to the lack of a therapeutic target, TNBC has a worse prognosis when compared to other molecular subtypes of breast cancer [4]. Therefore, the current therapeutic strategy for adjuvant therapy of TNBC is systemic chemotherapy, which can improve prognosis of TNBC patients.

Recently, there has been a steady increase in the incidence of T1N0 breast cancer due to accessible breast cancer screening programs in Korea [5,6]. Among patients with T1N0 breast cancers, the prevalence of patients with T1c stage is higher than that of patients with T1a or T1b stage [7]. Therefore, it is of clinical importance to determine the optimal treatment guidelines for patient with T1c stage TNBC breast malignancies.

Historically, the most utilized adjuvant chemotherapy regimen in Korea for T1N0 breast cancer was an adriamycin-containing regimen (either concurrent use of adriamycin plus cyclophosphamide [AC] or adriamycin/epirubicin plus cyclophosphamide plus 5-FU [FAC/FEC]), or a non-adriamycin containing regimen (including concurrent use of cyclophosphamide plus 5-FU plus methotrexate [CMF]). In the current national comprehensive cancer network (NCCN) guideline, there is a category 1 recommendation of adjuvant systemic chemotherapy including AC, FAC/FEC, or CMF regimens for node negative T1c TNBC patient. However, there are few clinical studies investigating the prognostic role of adjuvant chemotherapy in node negative T1c TNBC patient.

In this study, the prognostic role of adjuvant systemic chemotherapy in node negative, T1c TNBC breast cancer was investigated using a large cohort from the Korean breast cancer registry (KBCR). In addition, the prognostic effect between AC, FAC/FEC, and CMF regimens is compared in node-negative T1c breast cancer by subgroup analysis.

## Methods

### The Korean breast cancer registry

The KBCR is a nationwide database that includes patient data from 41 university hospitals, as well as 61 surgical training hospitals [8]. This database includes clinicopathological information including: patient survival, sex, age, surgical methods administered, pathological characteristics of the tumor, any adjuvant treatment received, and stage of cancer (based on the 7^th^ American Joint Committee on Cancer classification). Recently, survival information of patients in the KBCR was updated, with the last follow up date for patient survival recorded on December 31, 2014.

### Study cohort

From the KBCR database, 34,499 potential study patients who were diagnosed with breast cancer between May 1980 and December 2010 were retrospectively reviewed. Among them, a total of 2,217 patients with T1c N0 M0 TNBC were identified. In order to confirm the TNBC status of potential study patients, the immunohistochemical results for HER2 status were reviewed and the fluorescence in situ hybridization assay findings were used to confirm the HER2 status if the immunohistochemical results for HER2/neu staining were inconclusive (2+). After confirmation of TNBC status, patients were excluded if they had multifocal or multicentric breast cancer, history of previous ipsilateral or contralateral breast cancer, primary cancer histology other than invasive ductal subtype, or had undergone neoadjuvant systemic therapy. In addition, patients who were not treated with curative intent, those lacking follow-up data, or those without sufficient adjuvant treatment information, were also excluded. After applying the exclusion criteria, 1,151 patients remained for inclusion in this study. All patients who were treated with breast conserving surgery received adjuvant radiotherapy. This study was approved by the Institutional Review Board of St. Vincent Hospital. All participants in this study provided written informed consent for storage of their medical information in the database and use of this information in research. All experiments complied with the current laws of Korea.

### Patient treatment subgroups

Patients were categorized into four treatment groups dependent upon chemotherapy regimen: (1) no chemotherapy, (2) AC, (3) FAC/FEC, and (4) CMF. Patients in the AC group received 4 cycles of concurrent adriamycin + cyclophosphamide every 3 weeks. Patients in the FAC/FEC group received 6 cycles of concurrent 5-FU + Adriamycin/epirubicin + cyclophosphamide every 3 weeks. Patients in the CMF group received 6 cycles of concurrent 5-FU + methotrexate + cyclophosphamide every 4 weeks. The choice of systemic chemotherapy regimen was made at the clinician’s discretion, with all patients completing the full cycle of chemotherapy according to the standard chemotherapy protocol at the time.

### Statistical analyses

The following clinicopathological characteristics were included in the analysis: age at diagnosis, surgery type for breast and axilla, number of total harvested lymph node, tumor size, histologic grade, lymphovascular invasion, and information for adjuvant treatment (including systemic chemotherapy, radiotherapy). The primary endpoint of this study was overall survival (OS). OS was defined as the period from the date of breast cancer diagnosis until the date of death from any cause or the date of last follow-up. Clinicopathological characteristic differences between the treatment subgroups were compared using the independent *t* test, Pearson’s Chi-square test, or one-way analysis of variance. Survival curves were obtained using the Kaplan-Meier method, and significance was determined using the log-rank test. The multivariate analysis were conducted using Cox’s proportional hazard regression model for survival, with the hazard ratio (HR) and 95% confidence interval (95% CI) estimated for each variable. Multivariate models were adjusted for age at diagnosis, surgery type for breast and axilla, tumor size, histologic grade, lymphatic invasion, and systemic chemotherapy regimen. All statistical tests were two-sided, and the statistical significance was set at *p* < 0.05. All statistical analyses were performed using SPSS Statistics for Window, Version 12.0 (SPSS Inc., Chicago, IL, USA).

## Results

### Study cohort

Of the 1,151 patients included in the analysis, 1,006 received adjuvant chemotherapy while the remaining 145 received no chemotherapy. Among the 1,006 patients who received adjuvant chemotherapy, 586 received the AC regimen, 168 received the FAC/FEC regimen (42 FEC, 126 FAC), and 252 patients received the CMF regimen. The mean patient age was 49.11 ± 10.72 years (range, 22–80 years). The clinicopathological characteristics of the study cohort are shown in Table 1.

**Table 1:** Characteristics of the study cohort.

	No CTx.	AC		FAC/FEC		CMF		
	n = 145 (100%)	n = 586 (100%)	P value ^a)^	n = 168 (100%)	P value ^b)^	n = 252 (100%)	P value ^c)^	P value ^d)^
**Age (years)**	55.14 ± 13.05	47.57 ± 9.68	<0.001	46.51 ± 9.58	<0.001	50.95 ± 10.72	0.001	<0.001
**≤50**	58 (40.0%)	381 (65.0%)	<0.001	116 (69.0%)	<0.001	122 (48.4%)	0.105	<0.001
**>50**	87 (60.0%)	205 (35.0%)		52 (31.0%)		130 (51.6%)		
**Surgery**								
**BCS**	95 (65.5%)	488 (83.3%)	<0.001	141 (83.9%)	<0.001	147 (58.3%)	0.158	<0.001
**Mastectomy**	50 (34.5%)	98 (16.7%)		27 (16.1%)		105 (41.7%)		
**Axilla surgery**								
**ALND**	97 (66.9%)	330 (56.3%)	0.021	111 (66.1%)	0.905	218 (86.5%)	<0.001	<0.001
**SLND**	48 (33.1%)	256 (43.7%)		57 (33.9%)		34 (13.5%)		
**Total harvest LN**	8.92 ± 7.514	7.36 ± 5.625	0.02	7.18 ± 6.398	0.03	13.24 ± 9.729	<0.001	<0.001
**Tumor size (cm)**	1.593 ± 0.279	1.626 ± 0.277	0.198	1.652 ± 0.265	0.055	1.614 ± 0.277	0.473	0.368
**1<T±1.5**	77 (53.1%)	258 (44.0%)	0.05	67 (39.9%)	0.023	119 (47.2%)	0.259	0.331
**1.5<T±2**	68 (46.9%)	328 (56.0%)		101 (60.1%)		133 (52.8%)		
**Histologic grade**								
**G1**	12 (8.3%)	14 (2.4%)	<0.001	2 (1.2%)	<0.001	27 (10.7%)	0.375	<0.001
**G2**	53 (36.6%)	153 (26.1%)		28 (16.7%)		76 (30.2%)		
**G3**	80 (55.2%)	419 (71.5%)		138 (82.1%)		149 (59.1%)		
**Lymphatic invasion**								
**No**	129 (89.0%)	522 (89.1%)	0.969	119 (70.8%)	<0.001	223 (88.5%)	0.886	<0.001
**Yes**	16 (11.0%)	64 (10.9%)		49 (29.2%)		29 (11.5%)		
**Vascular invasion**								
**No**	134 (92.4%)	538 (91.8%)	0.811	159 (94.6%)	0.490	236 (93.7%)	0.637	0.374
**Yes**	11 (7.6%)	48 (8.2%)		9 (5.4%)		16 (6.3%)		
**Radiotherapy**								
**No**	47 (32.4%)	94 (16.0%)	<0.001	25 (14.9%)	<0.001	103 (40.9%)	0.094	<0.001
**Yes**	98 (67.6%)	492 (84.0%)		143 (85.1%)		149 (59.1%)		

Data are presented as mean ± SD or n (%)

CTx, chemotherapy; BCS, breast conserving surgery; ALND, axillary lymph node dissection; SLND, sentinel lymph node dissection; LN, lymph node.

a) P value for No CTx. vs. AC

b) P value for No CTx. vs. FAC/FEC

c) P value for No CTx. vs. CMF

d) P value for AC vs. FAC/FEC vs. CMF

Overall, patients receiving adjuvant chemotherapy were younger than patients who did not receive chemotherapy. In addition, there were more patients under 50 years old in the AC and FAC/FEC regimen groups compared to the no chemotherapy group. When compared to the no chemotherapy group, patients receiving the AC and FAC/FEC regimens were more likely to have a higher tumor histologic grade, received breast conserving surgery, and received adjuvant radiotherapy. The sentinel lymph node dissection was more likely to be performed in the no chemotherapy group than the CMF regimen group. In addition, the total number of harvested lymph nodes was highest in the CMF regimen group, followed by the no chemotherapy group, and then both the AC and FAC/FEC groups. Tumor size showed no significant difference between the no chemotherapy group and each of the regimen groups, but patients with a tumor size larger than 1.5 cm were more prevalent in the FAC/FEC group compared to the no chemotherapy group. Lymphatic invasion was more common in the FAC/FEC group than the no chemotherapy group. There was no significant difference in the occurrence of vascular invasion between the treatment groups.

The baseline clinicopathological characteristics were compared between each chemotherapy regimen groups. Generally, patients in the CMF group, when compared to other chemotherapy regimen groups, were older and more likely to have received a mastectomy, axillary lymph node dissection, and have a higher total number of harvested lymph nodes. By contrast, patients in the AC and FAC/FEC group, when compared to the CMF group, were more likely to have a higher tumor histologic grade as well as received adjuvant radiotherapy. Patients in the FAC/FEC regimen group exhibited more frequent lymphatic invasion than the other chemotherapy regimen groups. There was no significant difference found in either tumor size or occurrence of vascular invasion between the chemotherapy regimen groups.

### Survival outcomes for adjuvant chemotherapy

**Fig 1:**
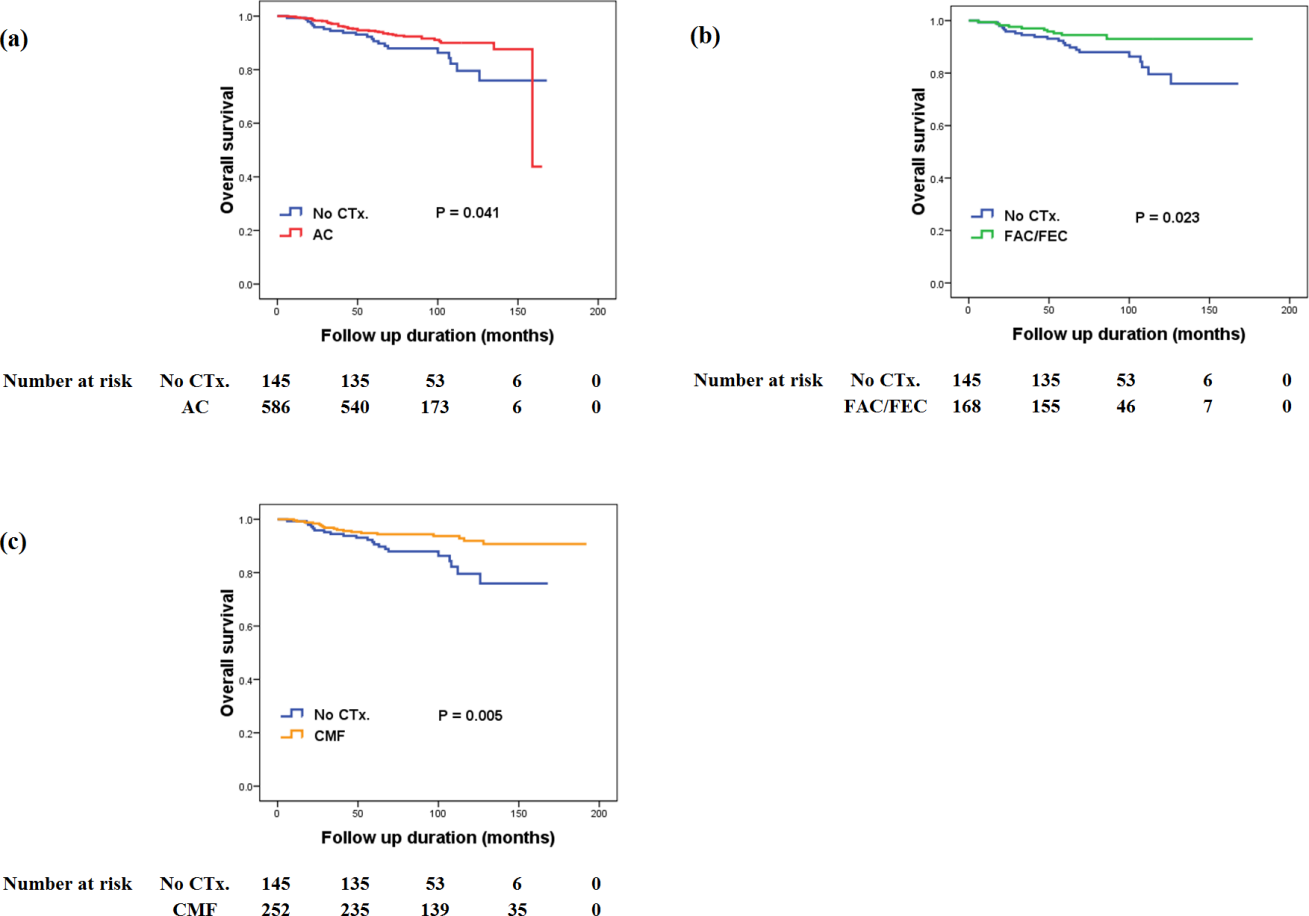
The association between adjuvant chemotherapy and overall survival. (a) No chemotherapy vs. AC regimen, (b) No chemotherapy vs. FAC/FEC regimen, (c) No chemotherapy vs. CMF regimen.

**Fig 2:**
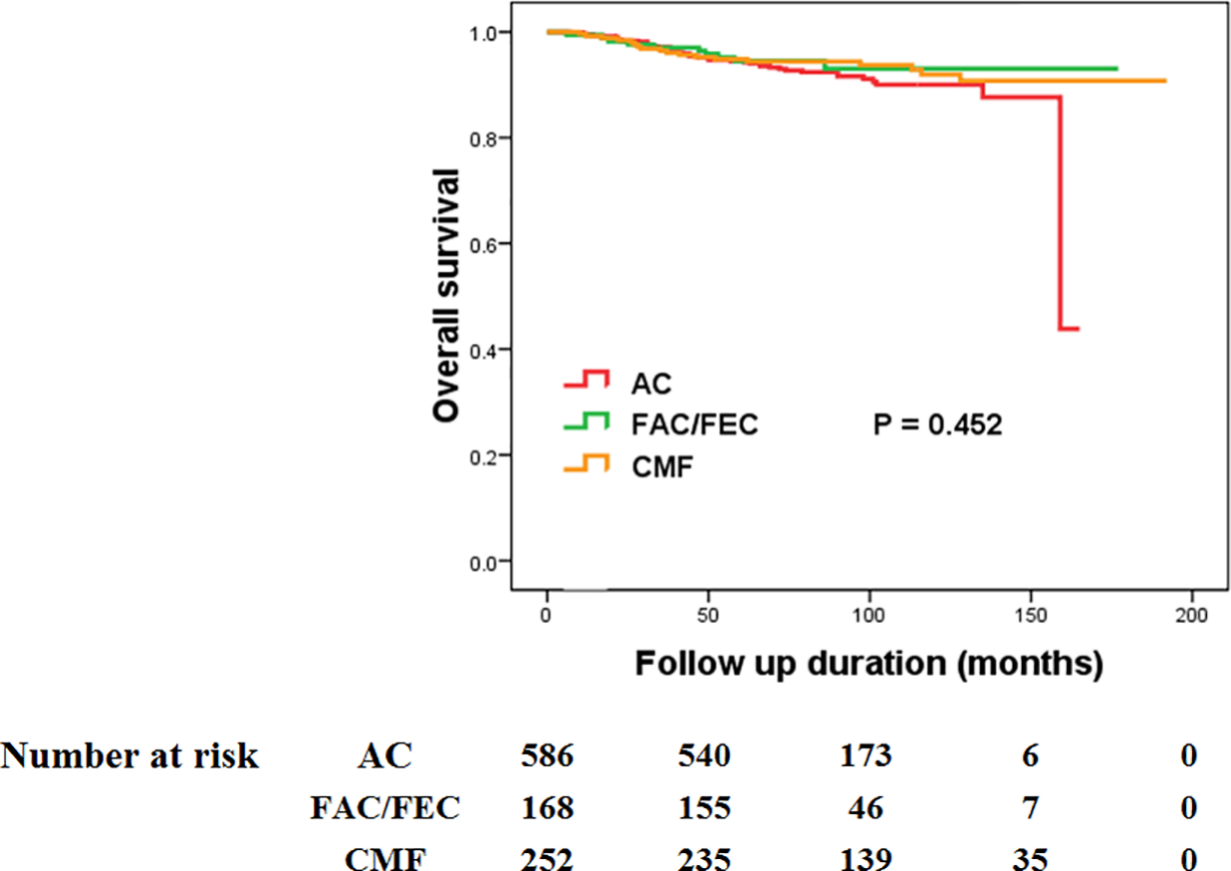
The comparison between chemotherapy regimens in overall survival (AC regimen vs. FAC/FEC regimen vs. CMF regimen).

The mean follow-up time of the study cohort was 87.98 ± 33.56 months (range, 6–192 months). During the follow-up period, the OS for study population was 91.66%, with death occurring in 96 patients. When the OS was analyzed between treatment regimens, patients in the no chemotherapy group showed significantly worse survival compared to all chemotherapy regimen groups: no chemotherapy vs. AC (85.5% vs. 92.0%, P = 0.041), no chemotherapy vs. FAC/FEC (85.5% vs. 94.0%, P = 0.023), and no chemotherapy vs. CMF (85.5% vs. 92.9%, P = 0.005) (Fig 1A–1C). However, when the OS was compared between each chemotherapy regimen group, there was no significant difference in OS between each chemotherapy regimen group (Fig 2).

### Multivariate analysis

To further investigate the prognostic effect of adjuvant chemotherapy on node-negative T1c TNBC patients, a multivariate analysis of OS was performed (Table 2). Patients in the no chemotherapy group consistently showed worse OS than each chemotherapy regimen group. However, there was no significant difference in OS between each chemotherapy regimen group.

**Table 2:** Multivariate analysis for overall survival.

No CTx.		AC		FAC/FEC		CMF	
HR (95% CI)	P value	HR (95% CI)	P value	HR (95% CI)	P value	HR (95% CI)	P value
1.000	Reference	0.587 (0.350–0.984)	0.043	0.427 (0.201–0.907)	0.027	0.438 (0.232–0.828)	0.011
–		1.000	Reference	0.639 (0.316–1.291)	0.212	0.678 (0.377–1.221)	0.196

CTx, chemotherapy; HR, hazard ratio; CI, confidence interval.

## Discussion

In this study, the prognostic role of adjuvant chemotherapy in T1c node negative TNBC patients was investigated. In addition, the difference of prognostic effect was investigated between several commonly prescribed adjuvant chemotherapy regimens for T1c node negative TNBC. As a result, adjuvant chemotherapy was found to be beneficial for OS in T1c node negative TNBC patients. Furthermore, this beneficial effect was observed in each chemotherapy regimen investigated in this study (AC, FAC/FEC, and CMF regimens).

Previous studies reported worse prognosis of the TN subtype compared to other molecular subtypes in node negative early stage breast cancer [9–11]. Therefore, in these patients, the importance of adjuvant systemic chemotherapy to improve prognosis could be more pronounced in TNBC than other molecular subtypes. In this regard, several previous studies reported the prognostic implication of adjuvant chemotherapy in node negative early stage TNBC patients. In 2010, Colleoni et al. report a beneficial prognostic effect of adjuvant chemotherapy in node negative TNBC patients [12]. Another study by Kim et al. that included 4,033 node negative T1–2 TNBC patients, also reported higher OS in patients receiving adjuvant chemotherapy compared to those not receiving adjuvant chemotherapy [13]. However, because these studies did not exclusively contain patients with T1 stage, but also those with T2 stage, the prognostic benefit of the adjuvant chemotherapy remains unclear for patients with node negative TNBC T1 stage disease.

Some studies reported a prognostic effect from adjuvant chemotherapy in subcategorized T1 stage patients with the node negative TNBC subtype. In 2013, Migdady et al. and Olszewski et al. reported that adjuvant chemotherapy did not significantly improve patient survival in node negative TNBC for stage T1a or T1b disease. [14,15]. Similarly, Vaz-Luis I et al. reported in 2014 that there was no significant prognostic effect of adjuvant chemotherapy in node negative TNBC patients, for either T1a or T1b stage [16]. Consequently, this lack of prognostic effect from adjuvant chemotherapy within the subcategorized T1 stage disease implies the need for further analysis separately between the T1a, T1b, and T1c stages.

The prognosis of T1c patients has been shown to be worse than T1a or T1b patients in previous studies. In 2009, Henry et al. report a higher recurrence rate in T1c patients compared to T1a for 110 node negative T1 TNBC subjects with a median 4.2 years follow-up (T1a 0% vs. T1c 8.2%) [17]. Additionally, Zachary et al. reported a lower disease-free survival of T1c as compared to T1mic, T1a, and T1b for 469 node negative T1 TNBC patients [18]. As a result of these studies, the current NCCN guideline strongly recommends adjuvant chemotherapy for T1c node negative TNBC patients because of their poor prognosis, despite no previous study exclusively investigating the prognostic effect of adjuvant chemotherapy for T1c node negative TNBC. To validate the current guideline, the prognostic effect of adjuvant chemotherapy was investigated exclusively in T1c node negative TNBC patients.

This was, to the best of our knowledge, the first study to investigate the prognostic effect of adjuvant chemotherapy in T1c node negative TNBC patients. There was one previous study that compared the prognostic effect between AC, FAC, and CMF regimens in node negative TNBC patients. In this study, Kim et al. reported a similar prognostic effect between AC, FAC, and CMF regimen in T1–2 node negative TNBC patients [13]. However, this study’s cohort was relatively heterogeneous in terms of breast cancer progression, including T stage. This heterogeneity could have skewed the perceived efficacy of each chemotherapy regimen. In 2014, Wu et al. report the effect on survival from adjuvant CMF chemotherapy regimen according to tumor size in node negative TNBC patients [19]. Therefore, it is plausible that the effect on survival by each chemotherapy regimen could differ between subcategorized tumor size subgroups for T1 node negative TNBC patients. In our study, a relatively homogenous study cohort was utilized and found similar prognostic effect between AC, FAC/FEC, and CMF regimens for T1c node negative TNBC. From the results of this study, there is evidence to verify the guidelines recommending adjuvant chemotherapy for T1c node negative TNBC patients.

This study had several limitations, which should be considered. First, this study was retrospectively designed. There exists the possibility of selection or information bias. Second, because the information for tumor recurrence and cause of death was not available in the recently updated KBCR database, survival could not be investigated in terms of tumor recurrence or breast cancer specific events. Third, despite of the recent use of taxane-based chemotherapy regimens for early breast cancer, no such patient group was included in this study.

## Conclusions

This study showed that adjuvant systemic chemotherapy may improve patient OS in T1c node negative TNBC, regardless of AC, FAC/FEC, or CMF chemotherapy regimen administered. A larger future study is needed to validate this study’s results.

## References

[1] Collins LC, Marotti JD, Gelber S, Cole K, Ruddy K, Kereakoglow S, et al Pathologic features and molecular phenotype by patient age in a large cohort of young women with breast cancer. Breast Cancer Res Treat 2012;131(3):1061–1066 doi: 10.1007/s10549-011-1872-9 22080245 10.1007/s10549-011-1872-9

[2] Kim JE, Ahn HJ, Ahn JH, Yoon DH, Kim SB, Jung KH, et al Impact of triple-negative breast cancer phenotype on prognosis in patients with stage I breast cancer. J Breast Cancer 2012;15(2):197–202 doi: 10.4048/jbc.2012.15.2.197 22807937 10.4048/jbc.2012.15.2.197 PMC3395743

[3] Choi J, Kim DH, Jung WK, Koo JS. Differential expression of immune-related markers in breast cancer by molecular phenotypes. Breast Cancer Res Treat 2013;137(2):417–429 doi: 10.1007/s10549-012-2383-z 23242618 10.1007/s10549-012-2383-z

[4] Lee JA, Kim KI, Bae JW, Jung YH, An H, Lee ES; Korean Breast Cancer Society. Triple negative breast cancer in Korea-distinct biology with different impact of prognostic factors on survival. Breast Cancer Res Treat 2010;123(1):177–187 doi: 10.1007/s10549-010-0998-5 20574671 10.1007/s10549-010-0998-5

[5] Park EH, Min SY, Kim Z, Yoon CS, Jung KW, Nam SJ, et al Basic Facts of Breast Cancer in Korea in 2014: The 10-Year Overall Survival Progress. J Breast Cancer 2017;20(1):1–11 doi: 10.4048/jbc.2017.20.1.1 28382089 10.4048/jbc.2017.20.1.1 PMC5378568

[6] Min SY, Kim Z, Hur MH, Yoon CS, Park EH, Jung KW; Korean Breast Cancer Society. The Basic Facts of Korean Breast Cancer in 2013: Results of a Nationwide Survey and Breast Cancer Registry Database. J Breast Cancer 2016;19(1):1–7 doi: 10.4048/jbc.2016.19.1.1 27066090 10.4048/jbc.2016.19.1.1 PMC4822102

[7] Ryu JM, Lee HJ, Yoon TI, Lee ES, Jung JH, Chae BJ, et al Breast cancer-specific mortality in small-sized tumor with node-positive breast cancer: a nation-wide study in Korean breast cancer society. Breast Cancer Res Treat 2016;159(3):489–498 doi: 10.1007/s10549-016-3943-4 27590199 10.1007/s10549-016-3943-4

[8] Moon HG, Han W, Hoh DY. Underweight and breast cancer recurrence and death: a report from the Korean Breast Cancer Society. J Clin Oncol 2009;17(35):5899–5905 10.1200/JCO.2009.22.4436 19805676

[9] Kwon JH, Kim YJ, Lee KW, Oh DY, Park SY, Kim JH, et al Triple negativity and young age as prognostic factors in lymph node-negative invasive ductal carcinoma of 1 cm or less. BMC Cancer 2010;10:557 doi: 10.1186/1471-2407-10-557 20946688 10.1186/1471-2407-10-557 PMC2966467

[10] Cancello G, Maisonneuve P, Rotmensz N, Viale G, Mastropasqua MG, Pruneri G, et al Prognosis in women with small (T1mic,T1a,T1b) node-negative operable breast cancer by immunohistochemically selected subtypes. Breast Cancer Res Treat 2011;127(3):713–720 doi: 10.1007/s10549-011-1465-7 21452022 10.1007/s10549-011-1465-7

[11] Gorshein E, Klein P, Boolbol SK, Shao T. Clinical significance of HER2-positive and triple-negative status in small (≤ 1 cm) node-negative breast cancer. Clin Breast Cancer 2014;14(5):309–314 doi: 10.1016/j.clbc.2014.02.007 24703318 10.1016/j.clbc.2014.02.007

[12] Colleoni M, Cole BF, Viale G, Regan MM, Price KN, Maiorano E, et al Classical cyclophosphamide, methotrexate, and fluorouracil chemotherapy is more effective in triple-negative, node-negative breast cancer: results from two randomized trials of adjuvant chemoendocrine therapy for node-negative breast cancer. J Clin Oncol 2010;28(18):2966–2973 doi: 10.1200/JCO.2009.25.9549 20458051 10.1200/JCO.2009.25.9549 PMC2982784

[13] Kim HA, Seong MK, Kim EK, Kang E, Park S, Hur MH, et al Evaluation of the Survival Benefit of Different Chemotherapy Regimens in Patients with T1-2N0 Triple-Negative Breast Cancer. J Breast Cancer 2015;18(3):271–278 doi: 10.4048/jbc.2015.18.3.271 26472978 10.4048/jbc.2015.18.3.271 PMC4600692

[14] Migdady Y, Sakr BJ, Sikov WM, Olszewski AJ. Adjuvant chemotherapy in T1a/bN0 HER2-positive or triple-negative breast cancers: application and outcomes. Breast 2013;22(5):793–798 doi: 10.1016/j.breast.2013.02.014 23489757 10.1016/j.breast.2013.02.014

[15] Olszewski AJ, Migdady Y, Boolbol SK, Klein P, Boachie-Adjei K, Sakr BJ, et al Effects of adjuvant chemotherapy in HER2-positive or triple-negative pT1ab breast cancers: a multi-institutional retrospective study. Breast Cancer Res Treat 2013;138(1):215–223 doi: 10.1007/s10549-013-2423-3 23354365 10.1007/s10549-013-2423-3

[16] Vaz-Luis I, Otteesen RA, Hughes ME, Mamet R, Burstein HJ, Edge SB, et al Outcomes by tumor subtype and treatment pattern in women with small, node-negative breast cancer: a multi-institutional study. J Clin Oncol 2014;32(20):2142–2150 doi: 10.1200/JCO.2013.53.1608 24888816 10.1200/JCO.2013.53.1608 PMC4076026

[17] Kaplan HG, Malmgren JA, Atwood M. T1N0 triple negative breast cancer: risk of recurrence and adjuvant chemotherapy. Breast J 2009;15(5):454–460 doi: 10.1111/j.1524-4741.2009.00789.x 19671105 10.1111/j.1524-4741.2009.00789.x

[18] Zumsteg ZS, Morrow M, Arnold B, Zheng Z, Robson M, Traina T, et al Breast-conserving therapy achieves locoregional outcomes comparable to mastectomy in women with T1-2N0 triple-negative breast cancer. Ann Surg Oncol 2013;20(11):3469–3476 doi: 10.1245/s10434-013-3011-9 23686101 10.1245/s10434-013-3011-9 PMC5730455

[19] Wu CE, Chen SC, Lin YC, Lo YF, Hsueh S, Chang HK. Identification of patients with node-negative, triple-negative breast cancer who benefit from adjuvant cyclophosphamide, methotrexate, and 5-fluorouracil chemotherapy. Anticancer Res 2014;34(3):1301–1306 24596377

